# Acute Bilateral Corneal Oedema Following Suspected Toxic Endothelial Insult: An Intriguing Case Report

**DOI:** 10.7759/cureus.113600

**Published:** 2026-07-29

**Authors:** Prerna Upadhyay, Shubha Rai, Nikita Shrivastava

**Affiliations:** 1 Ophthalmology, Sewa Sadan Eye Hospital, Bhopal, IND

**Keywords:** acute bilateral corneal oedema, alcohol-associated corneal oedema, corneal endothelial dysfunction, toxic endotheliopathy, toxic keratopathy

## Abstract

Visual recovery after a suspected toxic endothelial insult can be partial and uneven despite early improvement. We report a 40-year-old male who developed sudden bilateral blurring of vision with mild ocular discomfort after binge alcohol and marijuana intake, with an additional history of prolonged exposure to vehicle-emitted fumes. There was no history of trauma, contact lens use, ocular surgery, topical medication use, fever, discharge, or previous ocular disease. Examination showed severe bilateral visual reduction, epithelial oedema, diffuse stromal corneal oedema, central haze, and prominent Descemet’s membrane folds. The intraocular pressure was normal, and there was no evidence of infective keratitis, uveitis, acute glaucoma, or overt chemical injury. Pachymetry confirmed marked corneal thickening, while specular microscopy showed endothelial compromise, especially in the left eye. After excluding common causes, acute bilateral toxic endothelial dysfunction was considered. The patient was managed with a topical steroid-antibiotic combination, 5% hypertonic saline, and lubricants. Corneal oedema reduced gradually, with vision improving to 6/36 in both eyes by three days and further improvement on follow-up; however, persistent endothelial dysfunction remained in the left eye, requiring planned keratoplasty. This case emphasises that toxic endothelial dysfunction should be considered in sudden bilateral corneal oedema when typical signs of infection, inflammation, raised intraocular pressure, trauma, or chemical burn are absent.

## Introduction

Acute bilateral corneal oedema is an uncommon ophthalmic presentation characterised by a sudden loss of corneal transparency due to disruption of normal endothelial function. The corneal endothelium maintains corneal deturgescence through its barrier and pump mechanisms; even transient endothelial dysfunction may cause stromal hydration, Descemet membrane folds, and visual impairment. Because human corneal endothelial cells have limited regenerative capacity, significant endothelial injury may lead to persistent corneal oedema or decompensation [1]. Corneal oedema is commonly associated with Fuchs endothelial corneal dystrophy, intraocular surgery, raised intraocular pressure, infectious endotheliitis, trauma, or toxic endothelial injury [1,2].

Simultaneous bilateral acute corneal oedema is rare and should raise suspicion of systemic, environmental, metabolic, drug-related, or toxic causes rather than isolated unilateral ocular disease. Bilateral involvement suggests exposure to a common precipitating factor affecting endothelial function in both eyes. Reported causes include viral endotheliitis, drug-induced endothelial toxicity, plant toxin exposure, and chemical agents capable of impairing endothelial pump function [2,3].

Toxic endothelial dysfunction is an important but uncommon differential diagnosis in acute bilateral corneal oedema. Certain toxins may produce reversible corneal oedema by disrupting endothelial pump activity, particularly Na⁺/K⁺-ATPase-dependent fluid regulation, resulting in transient stromal hydration and sudden visual deterioration [3]. Recognition of this mechanism is clinically relevant because prompt exclusion of infectious, inflammatory, traumatic, and structural causes, along with supportive management, may result in complete visual recovery when endothelial injury is reversible. Published literature on acute bilateral corneal oedema following suspected toxic exposure remains limited, making such cases useful additions to clinical literature [3,4].

We report a case of acute bilateral corneal oedema with marked visual loss following suspected toxic endothelial insult, highlighting the diagnostic approach, exclusion of common causes, and favourable recovery pattern.

## Case presentation

A 40-year-old male presented to the ophthalmology outpatient department with complaints of sudden-onset blurring of vision in both eyes, associated with mild ocular discomfort for one day. He reported awakening in the morning with painless progressive haziness of vision affecting both eyes. There was no history of redness, ocular discharge, trauma, fever, contact lens wear, previous ocular surgery, or systemic illness. He denied any prior episodes of similar visual disturbance and had no known history of ocular disease.

On detailed personal history, the patient revealed daily consumption of alcohol and marijuana for approximately 10 years. He reported excessive intake of both substances on the night preceding the onset of symptoms. In addition, he recalled prolonged exposure to chemical fumes emitted from a truck that he had been closely following on the previous day. There was no history of use of topical ocular medications.

**Figure 1:**
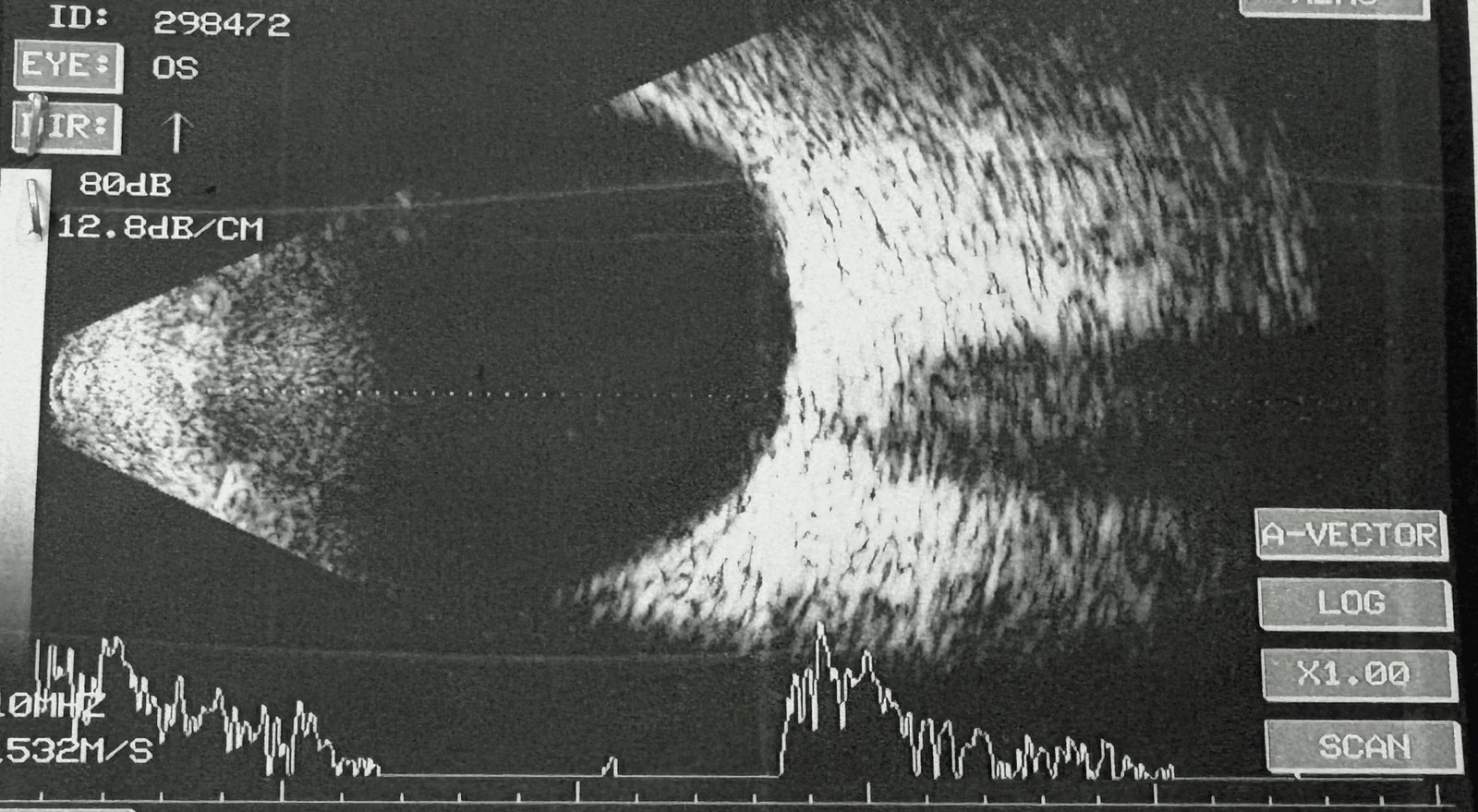
B-scan ultrasonography of the left eye showing a normal posterior segment.

On examination, best-corrected visual acuity was 2/60 in the right eye (RE) and counting fingers at 3 feet in the left eye (LE). Slit-lamp biomicroscopy revealed diffuse bilateral epithelial and corneal stromal oedema with central corneal haze involving the visual axis and prominent Descemet’s membrane folds. The corneal epithelium showed oedema with bullae in both eyes, with no epithelial defect, infiltrate, limbal ischaemia, or signs of chemical injury. There were no keratic precipitates, anterior chamber reaction, hypopyon, or evidence of uveitis. Pupillary reactions and extraocular movements were normal. Intraocular pressure was within normal limits in both eyes. Fundus examination was limited because of corneal haze. Ultrasonographic B-scan evaluation demonstrated no posterior segment abnormalities (Figure 1).

**Figure 2:**
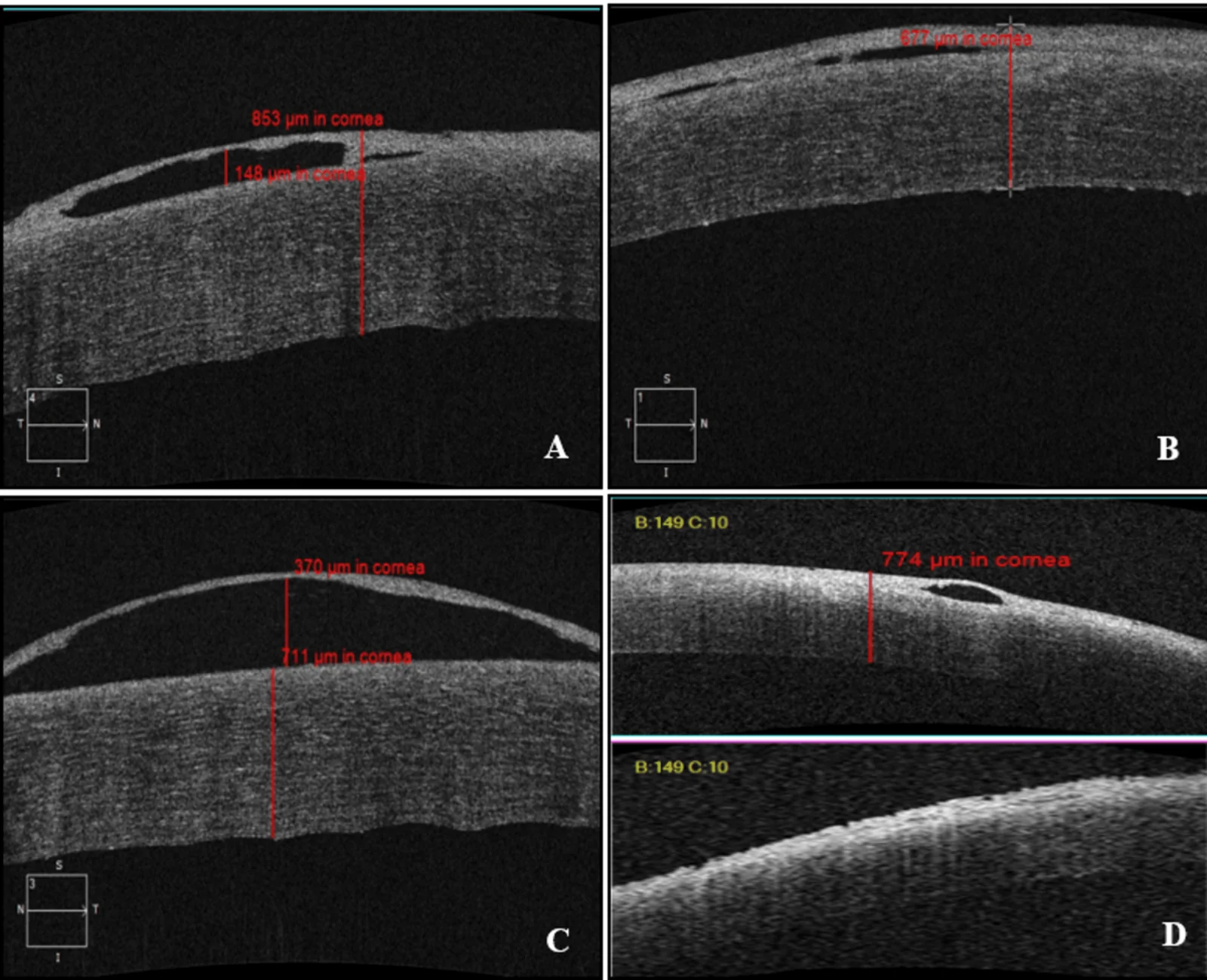
Serial anterior segment optical coherence tomography images showing bilateral corneal oedema with epithelial bullae and interval follow-up changes. (A) Right eye at presentation showing marked corneal oedema with an epithelial bulla. (B) Right eye at one-month follow-up showing reduction in corneal oedema; the hyperreflective foci observed along the endothelium are likely imaging artefacts or optical reflections related to the oedematous cornea rather than inflammatory cells. (C) Left eye at presentation showing marked corneal oedema. (D) Left eye at one-month follow-up demonstrating reduction in corneal oedema and decrease in the size of the epithelial bulla, with residual corneal thickening persisting.

Anterior segment optical coherence tomography and pachymetry confirmed marked corneal oedema with large epithelial bullae (Figure 2). The central corneal thickness was 677 μm in the RE and 711 μm in the LE. The bullous elevation was about 370 μm. Specular microscopy demonstrated an endothelial cell density of 1705 cells/mm² in the RE and 222 cells/mm² in the LE. Routine haematological and biochemical investigations were advised, which were unremarkable.

Based on the clinical presentation and history of recent excessive alcohol and marijuana consumption with possible inhalational toxic exposure, a provisional diagnosis of acute bilateral toxic corneal endothelial dysfunction was made. The patient was treated with topical prednisolone-antibiotic combination therapy, topical 5% hypertonic saline, topical 0.4% ripasudil, and lubricating eye drops containing 1% carboxymethylcellulose.

At the one-month follow-up, there was a gradual decrease in corneal oedema, and both eyes' visual acuity improved to 6/36. On subsequent follow-up, further resolution of corneal oedema was noted, with visual acuity improving to 6/18 in the RE and 6/36 in the LE. Despite clinical improvement, persistent endothelial dysfunction and residual corneal changes were evident in the LE. The patient was planned for Descemet stripping automated endothelial keratoplasty five months after the onset of symptoms; however, the patient was unfortunately lost to follow-up.

## Discussion

This case represents an unusual presentation of acute bilateral corneal oedema with marked endothelial dysfunction following binge alcohol intake, chronic alcohol and marijuana use, and possible inhalational exposure to vehicle-emitted chemical fumes. Bilateral acute corneal oedema in the absence of raised intraocular pressure, uveitis, epithelial defects, infiltrates, or limbal ischaemia is uncommon and should prompt consideration of transient endothelial pump failure or toxic endothelial injury.

Corneal endothelial dysfunction related to alcohol is rare. Reim et al. [5] first drew attention to alcohol-associated corneal changes, and Shiono et al. [6] later reported temporary bilateral corneal oedema after acute alcohol intake, with recovery of corneal clarity but persistent endothelial pleomorphism on follow-up. Similarly, Ranjan et al. [7] described acute bilateral toxic endotheliitis following alcohol consumption, where severe stromal oedema improved after intensive corticosteroid therapy. Olsen and Olsen [8] demonstrated a small but statistically significant transient increase in corneal thickness after ethanol ingestion in healthy volunteers and suggested temporary depression of endothelial pump activity as a possible mechanism.

The pathophysiology remains uncertain, but several mechanisms may be relevant. Ethanol and its metabolite acetaldehyde may exert direct toxic effects on the corneal endothelium and stromal proteins. Grütters et al. [9], in an in vitro study, observed reduced endothelial cell counts in corneas stored with acetaldehyde and increased alcohol-induced stromal protein degradation in corneas exposed to ethanol. Honey et al. [10] demonstrated a strong correlation between vitreous and blood alcohol concentrations in postmortem samples, supporting the possibility that high systemic alcohol levels may be reflected within ocular fluids. Therefore, elevated alcohol concentrations in the aqueous humour may plausibly impair endothelial pump function and contribute to stromal hydration.

Chronic alcohol use may further predispose the cornea to endothelial vulnerability. Sati et al. [11] reported significantly increased central corneal thickness and reduced endothelial cell density in patients with alcohol dependence syndrome compared with controls, with improvement after abstinence. Nutritional deficiency, hypoglycaemia, oxidative stress, and reduced antioxidant reserve may also contribute. The corneal endothelium is metabolically active and susceptible to oxidative injury; antioxidant vitamins have been shown experimentally to reduce oxidant-induced endothelial apoptosis [12]. In the present case, chronic alcohol use may have created a compromised endothelial reserve, while binge intake may have acted as an acute trigger.

In addition, the history of prolonged exposure to fumes emitted from a truck is clinically relevant. Kim et al. [13] reported acute bilateral corneal oedema after inhalation of spray paint despite absence of direct ocular chemical contact, with rapid resolution following topical corticosteroids and hypertonic saline. Experimental work by Vuong et al. [14] also showed that reactive chemical vapours, such as isocyanates, can induce corneal oedema and cytotoxic injury. Although our patient had no signs of classical chemical injury such as limbal ischaemia, epithelial defect, periocular burns, or mucosal involvement, inhalational toxic exposure may have contributed to endothelial dysfunction.

Viral endotheliitis was considered as a differential diagnosis; however, the absence of keratic precipitates, anterior chamber inflammation, raised intraocular pressure, recurrent episodes, or history suggestive of herpetic disease made it less likely. The rapid partial response to topical corticosteroids, hypertonic saline, and lubricants also supports a toxic or transient endothelial pump dysfunction rather than infective endotheliitis. The present case is notable because the clinical picture was dominated by bilateral corneal oedema with endothelial compromise, while features suggestive of infective keratitis, uveitis, acute glaucoma, or overt chemical burn were absent. In view of the temporal association with binge alcohol intake and possible fume exposure, a toxic endothelial insult was considered. However, causality cannot be established, and the event may represent transient endothelial pump dysfunction triggered by one or more toxic exposures in a susceptible cornea.

## Conclusions

This case illustrates severe bilateral corneal oedema as an unusual presentation of presumed toxic endothelial dysfunction. Toxic endothelial pump failure should be suspected in patients without indications of infection, uveitis, glaucoma, trauma or overt chemical harm. A detailed clinical history, especially about recent substance use and other environmental or occupational exposures, may provide valuable diagnostic clues when the underlying reason is not initially evident. Early detection, careful elimination of common causes, and supportive topical therapy may lead to meaningful improvement in vision. However, even with rapid treatment, recovery could not be complete, which calls for continuous follow-up to assess endothelial function and identify individuals who may eventually require endothelial keratoplasty.
